# Effect of Intracanal Irrigants on Coronal Fracture Resistance of Endodontically Treated Teeth Undergoing Combined Bleaching Protocol: An In Vitro Study

**Published:** 2018-09

**Authors:** Maryam Khoroushi, Sanaz Ziaei, Farinaz Shirban, Fatemeh Tavakol

**Affiliations:** 1 Professor, Dental Materials Research Center, Department of Operative Dentistry, Isfahan University of Medical Sciences, Isfahan, Iran; 2 Postgraduate Student of Pediatric Dentistry, School of Dentistry, Isfahan University of Medical Sciences, Isfahan, Iran; 3 Assistant Professor, Dental Research Center, Department of Orthodontics, School of Dentistry, Isfahan University of Medical Sciences, Isfahan, Iran; 4 Postgraduate Student of Oral Medicine, School of Dentistry, Tehran University of Medical Sciences, Tehran, Iran

**Keywords:** Root Canal Irrigants, Fracture, Endodontically-Treated Teeth, Tooth Bleaching Agents

## Abstract

**Objectives:**

Irrigation plays a critical role in endodontic treatment. Various single and combined irrigants and irrigation protocols are available. The aim of this study was to evaluate the effect of some common irrigation protocols on the coronal fracture resistance of endodontically treated teeth undergoing bleaching.

**Materials and Methods:**

After preparation of access cavities in 120 maxillary premolars, the teeth were divided into five groups (n=24) based on the irrigation protocol; G1:2.5% sodium hypochlorite (NaOCl), G2: 2% chlorhexidine (CHX), G3: NaOCl+CHX, G4: NaOCl+ethylenediaminetetraacetic acid (EDTA), G5: NaOCl+EDTA+CHX. Each group was subdivided into 2 subgroups of A: non-bleached (NB) and B: bleached (B). In subgroup B, the teeth underwent in-office and at-home bleaching techniques using 38% hydrogen peroxide and 20% carbamide peroxide gels for 3 weeks. The teeth were restored with composite resin, thermocycled, incubated for 24 hours, and underwent fracture resistance tests. Data were analyzed with one-way analysis of variance (ANOVA) and t-test (α=0.05).

**Results:**

T-test showed significant differences between every two corresponding groups (P<0.0001). In subgroup A, the minimum fracture resistance was recorded in G1. Also, G2 specimens exhibited a significantly higher fracture resistance compared to G1, G4, and G5. In subgroup B, G2 specimens exhibited a significantly higher fracture resistance compared to G1 and G4 that were irrigated using NaOCl and NaOCl+EDTA.

**Conclusions:**

It can be concluded that irrigation protocols can affect the coronal fracture resistance of bleached endodontically treated teeth, and specific irrigation protocols can be recommended for teeth undergoing bleaching.

## INTRODUCTION

Irrigation protocols play a critical role in sanitizing, shaping, and three-dimensional (3D) filling of the root canal system and are crucial for a successful endodontic therapy [1]. Many irrigants have been used for root canal preparation. The biocompatibility and antimicrobial and chemical activities of these solutions have been compared to select a perfect irrigant as an adjuvant to root canal therapy [2]. Various concentrations of sodium hypochlorite (NaOCl) are the most commonly used irrigation solutions. NaOCl, at 0.5%–5.25% concentrations, has two major applications including dissolving the pulpal tissue and destroying bacteria [3]. However, several recent reports have been issued on the adverse effects of NaOCl on the physical features of dentin such as flexural strength, elastic modulus, and microhardness [4–6]; the adverse effects have been ascribed to the changes in the inorganic and organic contents of dentin [7,8].

Ethylenediaminetetraacetic acid (EDTA) is a particular calcium-chelating agent, which is applied to eliminate the smear layer [9].

Chlorhexidine (CHX) is a biguanide irrigant used in endodontics. It has become popular due to its wide-spectrum antibacterial activity. However, it is unable to dissolve tissues [10].

Some recent surveys have assessed the discoloration potential of substances used during root canal treatment, including root canal irrigants, intracanal drugs, and endodontic filling materials [11]. Vivacqua-Gomes et al [12] noted dark brown deposits when NaOCl was mixed with CHX gel as a canal irrigant. The same discoloration was reported after the use of NaOCl with CHX solution [13]. Other studies have shown that interactions between CHX and EDTA irrigants result in the formation of white to pink deposits [14,15]. To achieve an aesthetic smile, discoloration is more important for many people than the normal alignment of the teeth within the arch. The aesthetic aspect of the teeth is of great significance for the general population and dental practitioners [11]. Internal bleaching is usually suggested for the teeth discolored following endodontic therapy [16].

Studies have reported the safety and effectiveness of external bleaching with a slow response [17,18]. However, some patients look for faster ways to get satisfactory results. Recently, combined bleaching techniques have been described for endodontically treated teeth, including in-office and at-home protocols. In external bleaching, the whitening agent is used on the labial/buccal surfaces of the teeth, while in internal bleaching, the whitening agent is placed within the pulp chamber [19].

A significant factor involved in the structural integrity of a restoration under occlusal forces is the fracture resistance of the restored tooth [20]. Recently, Indira and Nandlal [21] have reported that the use of CHX and NaOCl in disinfecting the cavity prepared for indirect composite inlays decreased the fracture resistance of the teeth. However, CHX resulted in a higher fracture resistance compared to NaOCl [21]. The impact of bleaching agents on the coronal fracture resistance of endodontically treated teeth is disputed by many researchers [22,23].

This study was undertaken to assess the effect of NaOCl, CHX, NaOCl+CHX, NaOCl+EDTA, and NaOCl+EDTA+CHX, as root canal irrigants, on the fracture resistance of endodontically treated teeth undergoing bleaching treatments.

The null hypothesis was that changes in irrigation protocols have no effect on the coronal fracture resistance of bleached teeth.

## MATERIALS AND METHODS

Following the approval by the Human Research Ethics Board of Isfahan University of Medical Sciences (#292179), 160 non-carious human premolars extracted for orthodontic reasons with similar crown and root sizes were selected. The premolars had two root canals with straight roots and mature apices without any calcification or resorption. By using a stereomicroscope (MBC-10, St. Petersburg, Russia) at 20× magnification, teeth with fractures and crack lines were excluded, and a total of 120 teeth were included in the study. The teeth were kept in 0.1% thymol solution at 4°C. In order to eliminate thymol residues, the teeth were stored in distilled water for 24 hours. Access cavities were prepared in the specimens using a tungsten carbide round-end fissure bur (Edenta AG, AU/SG, Switzerland) mounted on a high-speed handpiece with water coolant spray to eliminate the roof of the pulp chamber.

The working lengths were determined as 1 mm shorter than the root length using a #20 K-file (Mani Inc, Takanezawa Facility, Tochigi-Ken, Japan). Instrumentation was carried out with K-files up to a #60 instrument.

Afterwards, the teeth were randomly divided into five groups (n=24):

Group 1 (NaOCl): irrigation with 5 ml of 2.5% NaOCl (Ariadent, Asia ChemiTeb Co., Tehran, Iran) during instrumentation [24].
Group 2 (CHX): irrigation with 5 ml of 2% CHX (Merck KGaA, Darmstadt, Germany) during instrumentation [25].
Group 3 (NaOCl+CHX): irrigation with 5 ml of 2.5% NaOCl during instrumentation, followed by irrigation with 5 ml of distilled water for 1 minute and then with 5 ml of 2% CHX, as a final irrigant, for 1 minute [25].
Group 4 (NaOCl+EDTA): irrigation with 5 ml of 2.5% NaOCl during instrumentation, followed by irrigation with 5ml of distilled water for 1 minute and then with 5 ml of 17% EDTA (Ariadent, Asia ChemiTeb Co., Tehran, Iran), as a final irrigant, for 3 minutes [26,27].
Group 5 (NaOCl+EDTA+CHX): irrigation with 5 ml of 2.5% NaOCl during instrumentation, followed by irrigation with 5 ml of distilled water for 1 minute, irrigation with 17% EDTA at the end of instrumentation for 3 minutes and then with 5 ml of 2% CHX, as a final irrigant, for 1 minute. The canals were dried with paper points (Ariadent, Asia ChemiTeb Co., Tehran, Iran) and obturated using the lateral condensation technique with AH26 sealer (Dentsply DeTrey, Konstanz, Germany) and gutta-percha points (Ariadent, Asia ChemiTeb Co., Tehran, Iran).

Next, each group was divided randomly into two subgroups (n=12) of A: non-bleached (NB) and B: bleached (B).

In subgroup B, in order to place a cervical barrier, a heated plugger was used to remove 2 mm of gutta-percha, and a resin-modified glass-ionomer plug (Vitremer™, 3M ESPE, St. Paul, MN, USA) was inserted and light-cured (Demetron LC/Kerr, Danbury, CT, USA) for 40 seconds.

All the samples were embedded in a self-cured acrylic resin (Acropars, Marlic Medical Instruments CO., Tehran, Iran) in cylindrical molds (1.5×3.5 cm). The samples in subgroup B underwent bleaching. The whitening agents were 38% hydrogen peroxide (H_2_O_2_; Opalescence Xtra Boost, Ultradent Products Inc., South Jordan, UT, USA) and 20% carbamide peroxide (CH_6_N_2_O_3_; Ultradent Products Inc., South Jordan, UT, USA). The gels were applied to the entire buccal surfaces with a thickness of 0.5–1 mm and into the pulp chambers of the teeth. For the in-office bleaching technique, Opalescence Xtra Boost gel was used three times every 7 days. After 45 minutes, the gel was thoroughly removed using air-water spray. Between the in-office bleaching sessions, an at-home bleaching technique was carried out using 20% carbamide peroxide for two hours every day for three weeks. During the sessions, the gel was removed, and the surfaces were rinsed with distilled water. Between the sessions, all the samples were stored in artificial saliva at 37°C. After one week, the teeth were restored using the Single Bond etch-and-rinse adhesive system (3M ESPE, St. Paul, MN, USA) and composite resin (Clearfil AP-X, Kuraray Noritake Dental Inc., Tokyo, Japan) according to the manufacturers’ instructions (Table 1). A thermocycling procedure (Mp Based, KARA 1000 Inc, Tehran, Iran) consisting of 500 cycles at 5°/55°C with a 30-second dwell time and a 12-second transfer time was performed. Afterwards, the teeth were incubated at 37°C and 100% relative humidity for 24 hours. Finally, the specimens underwent a fracture resistance test in a universal testing machine (K-21546, Walterbai, Switzerland) as described below:

**Table 1.** Materials and their mode of application based on the manufacturers’ instructions

** Material **	** Mode of application **	** Manufacturer **
Vitremer	The Vitremer primer was applied for 30 seconds, air-dried for 15 seconds, and light-cured for 20 seconds. The Vitremer powder and liquid were mixed at a 2.5:1 ratio for 45 seconds. The paste was applied and light-cured for 40 seconds.	3M ESPE, St. Paul, MN, USA
Opalescence Xtra Boost	The activator syringe was mixed with the bleaching agent 10 times, and the mixture was expressed directly into the pulp chamber and onto the buccal surface for 45 minutes.	Ultradent Products Inc., South Jordan, UT, USA
Single Bond	The access cavity was etched with 35% phosphoric acid for 15 seconds, then rinsed with water spray for 10 seconds, leaving the tooth moist. Two consecutive coats of the adhesive were applied with a fully saturated brush tip and gently dried for 2–5 seconds, and light-cured for 10 seconds each.	3M ESPE, St. Paul, MN, USA
Clearfil AP-X	Composite resin was applied in 2-mm layers. Each layer was light-cured for 40 seconds.	Kuraray Noritake Dental Inc., Tokyo, Japan
Opalescence PF	The gel was applied in the access cavity and to the buccal surface of each specimen for 2 hours/day for 21 consecutive days.	Ultradent Products Inc., South Jordan, UT, USA
NaOCl	Irrigation protocol	Ariadent, Asia ChemiTeb Co., Tehran, Iran
EDTA	Irrigation protocol	Ariadent, Asia ChemiTeb Co., Tehran, Iran
CHX	Irrigation protocol	Merck, Germany

NaOCl=Sodium hypochlorite, EDTA=Ethylenediaminetetraacetic acid, CHX=Chlorhexidine

A round bar with a 5-mm diameter was positioned parallel to the long axis of each specimen and centered until it touched the slopes of the buccal and lingual cusps. A crosshead speed of 1 mm/minute was applied for a fracture to occur. The fracture was characterized by a sudden decrease in force and was measured in Newton (N). The data were analyzed by two-way analysis of variance (ANOVA), one-way ANOVA, and Tukey's honest significant difference (HSD) test at a significant level of 0.05. T-test was applied for pairwise comparisons.

The fractures were categorized as follows: 1) favorable fractures located more than 1 mm coronal to the cementoenamel junction (CEJ), and 2) unfavorable fractures located less than 1 mm coronal to the CEJ [28]. Fracture data were analyzed by Chi-square test.

## RESULTS

Although two-way ANOVA indicated the significant effect of irrigation protocols (P<0.001) and bleaching (P<0.001), considering the interaction effect P-value (P<0.001), oneway ANOVA and Tukey's test were applied.

The means, standard deviations (SD), and minimum/maximum values of fracture resistance in the groups are presented in Table 2.

**Table 2.** The means, standard deviations (SD), and minimum/maximum values of fracture resistance (Newton) in the groups

** Group **	** Bleaching **	** Mean **	** Std. Deviation **	** Std. Error **	** 95% CI for the Mean **		** Minimum **	** Maximum **

					** Lower Bound **	** Upper Bound **		
NaOCl (1)	NB	723	70	21	691	755	625	816
	B	403	101	35	321	485	255	595
CHX (2)	NB	1015	78	21	965	1066	910	1120
	B	511	79	23	461	561	390	600
NaOCl+CHX (3)	NB	914	70	29	863	965	810	1011
	B	499	71	20	453	544	350	576
NaOCl+EDTA (4)	NB	835	81	10	784	886	711	950
	B	378	79	23	328	430	222	496
NaOCl+EDTA+CHX (5)	NB	799	55	21	965	1066	910	1120
	B	495	107	31	427	563	360	672

NB=Non-Bleached, B=Bleached, CHX=Chlorhexidine, EDTA=Ethylenediaminetetraacetic acid, NaOCl=Sodium hypochlorite, CI=Confidence interval

T-test showed significant differences between every two corresponding groups (P<0.0001).

Subgroup A:
  The minimum fracture resistance was recorded in group 1 (NaOCl). The samples in group 2 (CHX) exhibited a significantly higher fracture resistance compared to groups 1, 4, and 5 (P<0.001). Group 3 showed a higher fracture resistance compared to group 1 (P<0.001). There were no significant differences between other groups (P>0.05).
Subgroup B:
The samples in group 2 (CHX) exhibited a significantly higher fracture resistance compared to groups 1 and 4 that were irrigated using NaOCl (P=0.049) and NaOCl+EDTA (P=0.036), respectively. There were no significant differences between other groups (P>0.05). Regarding the failure modes, the minimum rate of favorable fractures was observed in group 1 (NaOCl), However, there were no significant differences between the groups in terms of the fracture modes (Table 3).

**Table 3.** The frequency of failure modes

** Group **	** Favorable Fracture-NB **	** Favorable Fracture-B **	** Unfavorable Fracture-NB **	** Unfavorable Fracture-B **
** NaOCl N(%) **	5 (42)	4 (33)	7 (58)	8 (67)
** CHX N(%) **	6 (50)	6 (50)	6 (50)	6 (50)
** NaOCl+CHX N(%) **	8 (67)	7 (58)	4 (33)	5 (67)
** NaOCl+EDTA N(%) **	5 (42)	4 (33)	7 (58)	8 (33)
** NaOCl+EDTA+CHX N(%) **	8 (67)	8 (67)	4 (33)	4 (50)

NB=Non-Bleached, B=Bleached, CHX=Chlorhexidine, EDTA=Ethylenediaminetetraacetic acid, NaOCl = Sodium hypochlorite

## DISCUSSION

A review of the literature reveals the significant role of the clinicians who possess a thorough knowledge of the probable antagonistic interactions among endodontic irrigants commonly used in their clinical practice [29]. Since these solutions are utilized consecutively, they interact with each other within the intracanal space. This, in turn, may affect the treatment because of the changes in tissue dissolution, antimicrobial and cleaning efficacy, sealing, discoloration risk, and most importantly, the possible side effects to the patient’s general health as a result of leaching chemicals in periradicular tissues [29].

NaOCl is the only irrigant with the ability to dissolve the organic contents. Nonetheless, this irrigant is unable to remove the smear layer and should be used with other irrigants such as EDTA [30]. EDTA only removes the inorganic content of the smear layer, allowing direct contact with dentinal surfaces [31]

EDTA may influence the dentin structure, particularly dentin collagen, and it may have a potential of causing erosion in peritubular and intertubular dentin [31].

CHX is another endodontic irrigant with no capacity for eliminating the biofilm and other organic debris; therefore, use of NaOCl is necessary during root canal preparation [32]. It should be noted that the combination of NaOCl and CHX produces dark brown deposits [12,13]. 2% CHX might yield the best antibacterial effects at the final stage of chemomechanical preparations of root canals [33]. However, two different studies have shown the appearance of a white to light pink deposit subsequent to the interaction between CHX and EDTA [14,15]. Use of CHX alone can also lead to a dark brown discoloration of the tooth [34].

The results of the present study indicated that root canal irrigants can affect the coronal fracture resistance of endodontically treated teeth. Of all the irrigants evaluated in this study, the best fracture resistance test results were achieved with CHX.

In our study, NaOCl revealed the minimum fracture resistance in bleached and non-bleached subgroups. NaOCl can decrease the stiffness of the intertubular dentin matrix due to the heterogeneous distribution of the mineral phase within the collagen matrix; consequently, the hardness of dentin decreases [35]. NaOCl breaks down to oxygen and sodium chloride, oxidizing some components of the dentin matrix. As a result, the elastic modulus and flexural strength of the dentin diminish through the loss of organic materials [21,36]. The change in the dentin hardness might be an important factor in fracture resistance [37].

In the present study, irrigation with CHX alone or in combination with other solutions did not reduce the coronal fracture resistance. The microhardness of root canal dentin irrigated with different solutions, including 5.25% NaOCl, 2.5% NaOCl, 3% H_2_O_2_, 17% EDTA, and 0.2% CHX for 15 minutes each, showed that, except for CHX, all the irrigants decreased the dentin surface hardness [38]. Short-term applications of CHX at low concentrations prevented hybrid layer degradation and had a positive effect on the bond strength over time, consistent with previous findings [39].

A recent study on the effect of different irrigation protocols on the bond strength of fiber posts showed that use of 5.25% NaOCl in combination with 17% EDTA for post space irrigation can give rise to a decrease in bond strength, while use of 2% CHX as the final irrigant in this combination protocol can increase the bond strength [40]. This might be attributed to the ability of CHX to inhibit the matrix metalloproteinases (MMP) enzyme from decomposing the hybrid layer [40]. Another study evaluated the effect of CHX on the resin-dentin bond strength, demonstrating a better bond strength after 6 months of application of CHX [41].

In our study, irrigation with NaOCl and EDTA in bleached and non-bleached subgroups did not significantly decrease the fracture resistance of teeth since EDTA has the ability to remove the smear layer. Beltz et al [42] reported that EDTA has demineralization ability and can remove inorganic substances, altering the surface energy. Thus, the sealers flow easily in dentinal tubules, and the adhesion might be increased [42]. In the present study, using combination irrigation protocols in groups 3, 4, and 5 did not decrease the fracture resistance as much as the use of NaOCl. This can be attributed to the use of EDTA and CHX in these protocols.

Intracoronal tooth bleaching is usually recommended for teeth discolored consequent to endodontic treatment [16].

The bleaching technique used in this study comprised the use of 35% and 38% hydrogen peroxide as in-office and 10% and 20% carbamide peroxide as at-home bleaching agents in a combined bleaching protocol. [43]

In our study, subgroup B exhibited a lower fracture resistance compared to subgroup A. Studies have shown that internal bleaching with hydrogen peroxide can enhance dentin permeability [44], decrease the hardness of dentin and enamel [45], and weaken the dentin [46]. However, there is a disagreement about the effect of bleaching agents on the coronal fracture resistance of teeth [22,23]. The effect of combination bleaching protocol on decreasing the cusp fracture resistance has been shown previously [47]. The fracture resistance of endodontically treated teeth was not affected after 21 days of bleaching with 37% carbamide peroxide [23]. However, after two sessions of applying 38% hydrogen peroxide activated by a light-emitting diode (LED) laser system, the fracture resistance was reduced [22].

In terms of the failure modes, the lowest rate of favorable fractures was observed in subgroup 1 (NaOCl), which is rather correlated with the mean fracture resistance.

In this report, some of the protocols for canal irrigation during root canal treatment were evaluated, and only the coronal fracture resistance was studied. Other factors such as discoloration due to the application of different protocols should be considered during root canal treatment, especially in anterior teeth. Dentists should consider the effects of such protocols even when bleaching is applied as a combined treatment.

## CONCLUSION

Within the limitation of this study, it can be concluded that irrigation protocols used during endodontic treatment, with or without bleaching, can have different effects on the coronal fracture resistance. According to the results of this study, it could be suggested to use CHX alone or as a final irrigant in combination protocols for teeth undergoing a combined bleaching technique for a better coronal fracture resistance.

## References

[1-] Nair PN. On the causes of persistent apical periodontitis: a review. Int Endod J. 2006 4; 39 (4): 249– 81. 16584489 10.1111/j.1365-2591.2006.01099.x

[2-] Cintra LT Watanabe S Samuel RO da Silva Facundo AC de Azevedo Queiroz IO Dezan-Júnior E The use of NaOCl in combination with CHX produces cytotoxic product. Clin Oral Investig. 2014 4; 18 (3): 935– 40. 10.1007/s00784-013-1049-5 23892500

[3-] Kishen A. Mechanisms and risk factors for fracture predilection in endodontically treated teeth. Endod Topics. 2006 3; 13 (1): 57– 83.

[4-] Grigoratos D Knowles J Ng YL Gulabivala K. Effect of exposing dentine to sodium hypochlorite and calcium hydroxide on its flexural strength and elastic modulus. Int Endod J. 2001 3; 34 (2): 113– 9. 11307259 10.1046/j.1365-2591.2001.00356.x

[5-] Goldsmith M Gulabivala K Knowles JC. The effect of sodium hypochlorite irrigant concentration on tooth surface strain. J Endod. 2002 8; 28 (8): 575– 9. 12184417 10.1097/00004770-200208000-00003

[6-] Saleh AA Ettman WM. Effect of endodontic irrigation solutions on microhardness of root canal dentine. J Dent. 1999 1; 27 (1): 43– 6. 9922611 10.1016/s0300-5712(98)00018-9

[7-] Di Renzo M Ellis TH Sacher E Stangel I. A photoacoustic FTIRS study of the chemical modifications of human dentin surfaces: I. Demineralization. Biomaterials. 2001 4; 22 (8): 787– 92. 11246946 10.1016/s0142-9612(00)00240-4

[8-] Di Renzo M Ellis TH Sacher E Stangel I. A photoacoustic FTIRS study of the chemical modifications of human dentin surfaces: II. Deproteination. Biomaterials. 2001 4; 22 (8): 793– 7. 11246947 10.1016/s0142-9612(00)00239-8

[9-] Baumgartner JC Mader CL. A scanning electron microscopic evaluation of four root canal irrigation regimens. J Endod. 1987 4; 13 (4): 147– 57. 3106553 10.1016/s0099-2399(87)80132-2

[10-] Haapasalo M Shen Y Qian W Gao Y. Irrigation in endodontics. Dent Clin North Am. 2010 4; 54 (2): 291– 312. 20433979 10.1016/j.cden.2009.12.001

[11-] Ahmed HM Abbott PV. Discolouration potential of endodontic procedures and materials: a review. Int Endod J. 2012 10; 45 (10): 883– 97. 22621247 10.1111/j.1365-2591.2012.02071.x

[12-] Vivacqua-Gomes N Ferraz CC Gomes BP Zaia AA Teixeira FB Souza-Filho FJ. Influence of irrigants on the coronal microleakage of laterally condensed gutta-percha root fillings. Int Endod J. 2002 9; 35 (9): 791– 5. 12449031 10.1046/j.1365-2591.2002.00569.x

[13-] Basrani BR Manek S Sodhi RN Fillery E Manzur A. Interaction between sodium hypochlorite and chlorhexidine gluconate. J Endod. 2007 8; 33 (8): 966– 9. 17878084 10.1016/j.joen.2007.04.001

[14-] Rasimick BJ Nekich M Hladek MM Musikant BL Deutsch AS. Interaction between chlorhexidine digluconate and EDTA. J Endod. 2008 12; 34 (12): 1521– 3. 19026886 10.1016/j.joen.2008.08.039

[15-] Gonzalez-Lopez S Camejo-Aguilar D Sanchez-Sanchez P Bolanos-Carmona V. Effect of CHX on the decalcifying effect of 10% citric acid, 20% citric acid, or 17% EDTA. J Endod. 2006 8; 32 (8): 781– 4. 16861082 10.1016/j.joen.2006.02.006

[16-] Roberto AR Sousa-Neto MD Viapiana R Giovani AR Souza Filho CB Paulino SM Effect of different restorative procedures on the fracture resistance of teeth submitted to internal bleaching. Braz Oral Res. 2012 Jan-Feb; 26 (1): 77– 82. 22344342 10.1590/s1806-83242012000100013

[17-] Barghi N. Making a clinical decision for vital tooth bleaching: at-home or in-office? Compend Contin Educ Dent. 1998 8; 19 (8): 831– 8. 9918107

[18-] Leonard RH Jr Bentley C Eagle JC Garland GE Knight MC Phillips C. Nightguard vital bleaching: a long-term study on efficacy, shade retention, side effects, and patients’ perceptions. J Esthet Restor Dent. 2001; 13 (6): 357– 69. 11778855 10.1111/j.1708-8240.2001.tb01021.x

[19-] Plotino G Buono L Grande NM Pameijer CH Somma F. Nonvital tooth bleaching: a review of the literature and clinical procedures. J Endod. 2008 4; 34 (4): 394– 407. 18358884 10.1016/j.joen.2007.12.020

[20-] Mahler DB. Research on dental amalgam: 1982–1986. Adv Dent Res. 1988 8; 2 (1): 71– 82. 3073786 10.1177/08959374880020012301

[21-] Indira MD Nandlal B. Comparative evaluation of the effect of cavity disinfectants on the fracture resistance of primary molars restored with indirect composite inlays: an in vitro study. J Indian Soc Pedod Prev Dent. 2010 Oct-Dec; 28 (4): 258– 63. 21273713 10.4103/0970-4388.76153

[22-] Pobbe Pde O Viapiana R Souza-Gabriel AE Marchesan MA Sousa-Neto MD Silva-Sousa YT Coronal resistance to fracture of endodontically treated teeth submitted to light-activated bleaching. J Dent. 2008 11; 36 (11): 935– 9. 18771836 10.1016/j.jdent.2008.07.007

[23-] Bonfante G Kaizer OB Pegoraro LF do Valle AL. Fracture resistance and failure pattern of teeth submitted to internal bleaching with 37% carbamide peroxide, with application of different restorative procedures. J Appl Oral Sci. 2006 8; 14 (4): 247– 52. 19089271 10.1590/S1678-77572006000400007 PMC4327481

[24-] Radcliffe CE Potouridou L Qureshi R Habahbeh N Qualtrough A Worthington H Antimicrobial activity of varying concentrations of sodium hypochlorite on the endodontic microorganisms Actinomyces israelii, A. naeslundii, Candida albicans and Enterococcus faecalis. Int Endod J. 2004 7; 37 (7): 438– 46. 15189432 10.1111/j.1365-2591.2004.00752.x

[25-] Gomes BP Souza SF Ferraz CC Teixeira FB Zaia AA Valdrighi L Effectiveness of 2% chlorhexidine gel and calcium hydroxide against Enterococcus faecalis in bovine root dentine in vitro. Int Endod J. 2003 4; 36 (4): 267– 75. 12702121 10.1046/j.1365-2591.2003.00634.x

[26-] Scelza MF Pierro V Scelza P Pereira M. Effect of three different time periods of irrigation with EDTA-T, EDTA, and citric acid on smear layer removal. Oral Surg Oral Med Oral Pathol Oral Radiol Endod. 2004 10; 98 (4): 499– 503. 15472668 10.1016/j.tripleo.2004.03.027

[27-] Heling I Chandler NP. Antimicrobial effect of irrigant combinations within dentinal tubules. Int Endod J. 1998 1; 31 (1): 8– 14. 9823123

[28-] Uyehara MY Davis RD Overton JD. Cuspal reinforcement in endodontically treated molars. Oper Dent. 1999 Nov-Dec; 24 (6): 364– 70. 10823086

[29-] Rossi-Fedele G Doğramacı EJ Guastalli AR Steier L de Figueiredo JA. Antagonistic interactions between sodium hypochlorite, chlorhexidine, EDTA, and citric acid. J Endod. 2012 4; 38 (4): 426– 31. 22414823 10.1016/j.joen.2012.01.006

[30-] Khademi A Feizianfard M. The effect of EDTA and citric acid on smear layer removal of mesial canals of first mandibular molars, a scanning electron microscopic study. J Res Med Sci. 2004 4; 9 (2): 80– 8.

[31-] Wagner MH da Rosa RA de Figueiredo JAP Duarte MAH Pereira JR Só MVR. Final irrigation protocols may affect intraradicular dentin ultrastructure. Clin Oral Investig. 2017 9; 21 (7): 2173– 2182. 10.1007/s00784-016-2006-x 27888349

[32-] Buck RA Eleazer PD Staat RH Scheetz JP. Effectiveness of three endodontic irrigants at various tubular depths in human dentin. J Endod. 2001 3; 27 (3): 206– 8. 11487153 10.1097/00004770-200103000-00017

[33-] Zamany A Safavi K Spångberg LS. The effect of chlorhexidine as an endodontic disinfectant. Oral Surg Oral Med Oral Pathol Oral Radiol Endod. 2003 11; 96 (5): 578– 81. 14600693 10.1016/s1079-2104(03)00168-9

[34-] Sulieman M. An overview of tooth discoloration: extrinsic, intrinsic and internalized stains. Dent Update. 2005 10; 32 (8): 463– 4, 466– 8, 471. 16262034 10.12968/denu.2005.32.8.463

[35-] Jhamb S Nikhil V Singh V. Effect of sealers on fracture resistance of endodontically treated teeth with and without smear layer removal: An in vitro study. J Conserv Dent. 2009 Jul-Sep; 12 (3): 114– 7. 20543918 10.4103/0972-0707.57635 PMC2879718

[36-] Prabhakar AR Hadakar SG Raju OS. Comparative evaluation of pH and antibacterial effect of various calcium hydroxide combinations on E. faecalis and its effect on root strength: An in vitro study. Contemp Clin Dent. 2012 1; 3 (1): 42– 7. 22557896 10.4103/0976-237X.94545 PMC3341758

[37-] Arslan H Barutcigil C Karatas E Topcuoglu HS Yeter KY Ersoy I Effect of citric acid irrigation on the fracture resistance of endodontically treated roots. Eur J Dent. 2014 Jan-Mar; 8 (1): 74– 8. 24966750 10.4103/1305-7456.126248 PMC4054036

[38-] Marshall GW Jr Balooch M Kinney JH Marshall SJ. Atomic force microscopy of conditioning agents on dentin. J Biomed Mater Res. 1995 11; 29 (11): 1381– 7. 8582906 10.1002/jbm.820291109

[39-] Leitune VC Portella FF Bohn PV Collares FM Samuel SM. Influence of chlorhexidine application on longitudinal adhesive bond strength in deciduous teeth. Braz Oral Res. 2011 Sep-Oct; 25 (5): 388– 92. 22031050 10.1590/s1806-83242011000500003

[40-] Ertas H Ok E Uysal B Arslan H. Effects of different irrigating solutions and disinfection methods on push-out bond strengths of fiber posts. Acta Odontol Scand. 2014 11 1; 72 (8): 783– 7. 24773130 10.3109/00016357.2014.906651

[41-] Yu W Guo K Zhang B Weng W. Fracture resistance of endodontically treated premolars restored with lithium disilicate CAD/CAM crowns or onlays and luted with two luting agents. Dent Mater J. 2014; 33 (3): 349– 54. 24739817 10.4012/dmj.2013-240

[42-] Beltz RE Torabinejad M Pouresmail M. Quantitative analysis of the solubilizing action of MTAD, sodium hypochlorite, and EDTA on bovine pulp and dentin. J Endod. 2003 5; 29 (5): 334– 7. 12775005 10.1097/00004770-200305000-00004

[43-] Basting RT Amaral FL França FM Flório FM. Clinical comparative study of the effectiveness of and tooth sensitivity to 10% and 20% carbamide peroxide home-use and 35% and 38% hydrogen peroxide in-office bleaching materials containing desensitizing agents. Oper Dent. 2012 Sep-Oct; 37 (5): 464– 73. 22616927 10.2341/11-337-C

[44-] Carrasco LD Fröner IC Corona SA Pécora JD. Effect of internal bleaching agents on dentinal permeability of non-vital teeth: quantitative assessment. Dent Traumatol. 2003 4; 19 (2): 85– 9. 12656839 10.1034/j.1600-9657.2003.00112.x

[45-] Chng HK Ramli HN Yap AU Lim CT. Effect of hydrogen peroxide on intertubular dentine. J Dent. 2005 5; 33 (5): 363– 9. 15833391 10.1016/j.jdent.2004.10.012

[46-] Chng HK Palamara JE Messer HH. Effect of hydrogen peroxide and sodium perborate on biomechanical properties of human dentin. J Endod. 2002 2; 28 (2): 62– 7. 11837248 10.1097/00004770-200202000-00003

[47-] Khoroushi M Feiz A Khodamoradi R. Fracture resistance of endodontically-treated teeth: effect of combination bleaching and an antioxidant. Oper Dent. 2010 Sep-Oct; 35 (5): 530– 7. 20945744 10.2341/10-047-L

